# Dr. Billing on the First Principles of Medicine

**Published:** 1831-04-01

**Authors:** 


### XIII.

**First Principles of Medicine.** By *Archibald Billing*, M.D. Fellow of the Royal College of Physicians, Lecturer on Medicine at the London Hospital, &c. 8vo. pp. 131. Underwoods, 1831.

Although this little volume appears principally designed for students, a perusal of its contents has led us to form a very favourable opinion both of the author and the work. It is justly observed that "the object of lectures is to convey to the student, in a condensed manner, that knowledge, in abstract, which will enable him to understand what he sees at the bed-side, and the observations of the clinical professor;—without which clinical instruction all that the memory may be charged with from books or lectures, is but vanity." It is a great pity that the medical officers of our hospitals and infirmaries are only just now becoming alive to this truth—probably from a consciousness that their own personal and pecuniary interests are at stake on the occasion! We shall now proceed to give some extracts from this little volume which will, we think, confirm the favourable opinion which we expressed of it in the outset.

### The Circulation.

"The heart *contracts* by *muscular* action and *relaxes* alternately, the arteries keep up a constant *contractile pressure* on their contents, not an alternate contraction and relaxation, but a continued contractile effort which is overcome by the action of the heart; when there is much blood sent into them they are distended, and if there be little blood sent into them, as after hæmorrhage, their tendency to contract causes them to close, so as to keep always full, and to preserve a constant stream of the blood even during the temporary relaxation of the heart; and the arteries yielding and adapting themselves to the pressure of the heart and recontracting on their contents, whilst the heart is relaxed and filling, is the cause of the equability of the stream in the veins, nay, the stream even in the arteries is much less in jets than is supposed by those who judge from the mode of its flowing from a wounded artery; for though, when there is a free escape from the wound, the impulse of the heart causes an unequal stream, it must be remembered that in the tube, unwounded, that force would have been expended in stretching the artery, whereas the artery when wounded, ceases to be other than as a simple tube, the elasticity not being called into operation, on account of the escape of the blood from the wound.

The most simple mechanical illustration perhaps, is the double bellows of a smith's forge, which keeps up a constant current of air; though the handle works with intermissions, so that the blast into the fire would be in puffs, if it were not for the weight of the upper half of the bellows, which keeps forcing out the air in a continued current, whilst the hand is drawing back to make another impulse.

It has been supposed that the circular fibres of the ARTERIES were muscular, and that they contracted and relaxed at each pulse, and that the throb felt, was caused by a dilatation of the artery; those fibres are NOT MUSCULAR, but more approaching to a ligamentous tissue, firm and, though elastic, not yielding to the force of the heart, but on the contrary, preserving the CALIBRE of the artery UNIFORM, as may be seen by laying bare an artery in a living animal, or when the artery is laid bare in an operation; it is LONGITUDINALLY that the arteries are STRETCHED at each injection from the heart, by which their capacity is increased, the consequence of which, from their being bound down in various places, is, that there is a SERPENTINE motion in the artery where it is at all loose.

The fibres of the middle coat of the artery being arranged circularly, allow of the separation laterally, and thus *accommodate* themselves to the *elongation*, of the tube, whilst they *resist* its *dilatation*. Now it may be thought that the movements of arteries *seen* at the wrist and in the temples is their dilatation, but it is the serpentine motion caused by the alteration of the curve, the artery being elongated at each injection from the heart.

Where the artery is perfectly straight you may lay it bare and scarcely see it move, but the moment you compress it with the finger, or tie a ligature around it, you perceive it pushed at every pulse. To illustrate the deception of the sensation which the pulse gives, as if the artery were dilated at each beat; if a long vein removed from the body have a syringe adapted to one end, the other being raised, or arranged with a spring valve, which yields to the jets so as to keep it full, and fluid be sent through in jets, it will upon pressure by the finger give the sensation of dilatation, but the eye perceives none: again, if any one grasp the leather tube of a fire or garden engine, the sensation given will be that of its expanding in the hand at each stroke of the pump, but the eye contradicts the sensation, it is merely the tendency to resume the cylindrical form from the outward pressure of the fluid, but not expansion.

It has been *attempted* to be *proved* that the heart has an *active power of dilatation*,* by which it helps to *refill* itself, sucking in the blood as it were, and one proof is brought forward from the heart of large mammalia, as the horse, ox, or whale, which affords the phenomenon of contracting and expanding after removal from the body; but the expansion is simple relaxation, as when after the fist has been shut with an effort, upon the relaxation of the flexor muscles it will be seen to expand slightly without any action of the extensors; and when a large heart is relaxing, if the hands be pressed on opposite sides of it they will be sensible of an apparently active expansion from the mere gravitating recovery

---

* "The '*impulsion*' of the heart against the side (which takes place between the first and second sound, that is just as the auricles have filled the ventricles, and the latter become rigid commencing their contraction), is in proportion to its muscular action, and is produced by the heart assuming a form more approaching to the globular, and becoming firm at the same time, as when the gastrocnemii muscles act; the heart being in an angle between the diaphragm and parietes of the chest, the increase of its transverse dimensions has the effect of the driving of a wedge, forcing against the ribs. Now these actions may be very forcible, but if they be too rapid, there is not time for the heart to receive the usual quantity, so that only a little being sent into the arteries at each contraction, the pulse may be small, when the heart is acting strongly, as in palpitations, and an ignorance of this fact might lead to the administration of stimulants when not required (to say the least), if the pulse alone were consulted; cæteris paribus impulsion is increased by hypertrophy, diminished by dilatation."

of position of such a mass of matter. The heart, in fact, not opening, but *falling open after* each *contraction*." 12.

The above is probably as good an explanation of the circulation as our present state of knowledge can offer. It will be seen that our author has not adopted the theory of Dr. Corrigan on this occasion.

Passing over the section on "nervous influence," a subject that has hitherto baffled all researches, we come to inflammation; and it will be seen, by the following extract, that Dr. Billing has adopted the more rational opinion that the vessels are in a state of *dilatation* rather than morbid *activity*, where inflammation obtains.

"An inflamed part is redder and swelled; where the vessels are visible, as in the eye, we can see the redness is caused by the minute vessels becoming larger, so as to admit red globules of the blood, which before admitted only the more fluid and transparent part, or the minute vessels carrying red globules becoming larger; this enlargement of vessels is not from increased action, but on the contrary, from their action being diminished, their giving way, and being *dilated* by the injecting force of the heart. The way to diminish the inflammation is by increasing the action of the arteries, as by cold or astringents, which make the arteries contract, that is, increases their action; so that so far from the arteries in an inflamed part being in a state of increased action, one of the means of *diminishing inflammation* is by *increasing* arterial *action* in the part inflamed. It is common to remark the *throbbing* of the *carotid* arteries as increased action, but the more they throb it shews that they the *more yield* to the injecting force of the heart; when the eye, or any other part, is injured by heat, or a stream of cold air, a blow, or cantharides plaister applied to the skin, &c. the part becomes redder from the vessels enlarging, and carrying either more red blood, or admitting red blood where there was none before. Now in this first and simplest instance of inflammation, the heart does not act more strongly than ordinary, not affecting the pulse, so that the capillary arteries evince debility, having given way when there is no more force than they bore before without distention; from this they sometimes recover of themselves, gradually contracting to their natural size; or if not, the simple application of cold, or astringent lotion, makes them contract, and the redness disappears.

On the other hand, by savine or cantharides ointment we can produce an inflammation, such a relaxation of the capillaries that those which can be dilated, are, and separate from those which are confined by firm surrounding substance, by which means warts are thrown off, which has been called increasing vascular action beyond what they could bear; it is increasing the *size* of the vessels, but not their *action*. It is the opinion of some persons even at the present day, that the motion of the *blood* is *accelerated* in inflamed parts, though the experiments of Parry and others prove the *contrary* to be the case, as follows from the *capillaries* being *enlarged*, inasmuch as when fluid passes through a given space, the current beyond that will be slower in proportion to the wideness of the channel, as every one must have observed in a wide part of a river, where the current becomes slower; and the same may be observed by passing water mixed with grains of amber through a glass tube with a bulbous enlargement in the middle, the current will slacken in the bulb and resume its velocity beyond it.

Some will allow that the capillary arteries where the blush of inflammation is are weak, as they visibly have given way, but they still talk of *increased arterial action*, and say that the arteries *around* or leading to the inflamed part are in increased activity, as a part of the condition or what keeps up the inflammation; not considering that an increase in their action would be contraction, and so a diminution of the flow of blood to the inflamed part; wherefore an increased action in the arteries both in and leading to the inflamed part is just what is required to diminish the inflammation.

The more the heart acts the more of course it forces the arteries of the inflamed part and the *pulse* shewing the degree of power of *action* of the *heart* is erroneously by some considered as an evidence of arterial action, as the throbbing of the carotid arteries for instance ; the heart then acting against the capillaries, if we cannot get them to act strongly enough to resist its force we are obliged to diminish the force of the circulation, either by taking away blood which diminishes both the quantity of blood sent to the arteries and the action of the heart itself, and in this way we leave less for the arteries of the inflamed part to do ; or, we can lower the force of the heart by medicines, such as digitalis, &c. ; here for illustration the simplest cases of inflammation have been taken, where the heart is acting naturally the inflammation being from injury." 23.

Dr. Billing takes occasion to pass a well-merited eulogium on M. Laennec. "Compared (says he) with what we know of diseases of the viscera in the chest, the sum of information attained twenty years ago, was but darkness visible."

We cannot follow our author through all his disquisitions, which are generally ingenious and practical ; but must content ourselves with one or two more extracts.

" I may now offer some illustrations of the effect of stimulants, sedatives, and narcotics, singly in producing good results.

A boy was brought into the London Hospital with a swelling of the knee, which was in constant pain, he had been in some days when I saw him, he was much emaciated, and irritable and languid, consumed by hectic feverishness, got no refreshing sleep from opiates, the pulse 130, thready or rather wiry, very hard, he was too weak to apply more leeches to the knee, though hot, tender to the touch, and not bearing the slightest motion, as he kept it constantly bent, and could not bear it moved ; the indication was to take off the injecting force as the vessels could not be otherwise relieved ; fifteen drops of tincture of digitalis were ordered three times in the twenty-four hours ; after the second dose he got better sleep than he had from the opiates, the pulse came down, and in less than a fortnight he grew stouter, as the knee grew smaller, and was able to walk home.

A medical student had swelled knee with great pain preventing rest, he was treated secundem artem, and amongst the rest by one of the best surgeons now living in England, he had not fever, did not emaciate, but was tormented with pain, and sometimes painful applications ; one night, coute qui coute, he took what he imagined to be about a tea-spoonful out of a bottle of tincture of opium ; he slept twelve or fourteen hours, awoke free from pain, and very soon walked to the hospital without any more medicine.

Instead of a knee case, cured by stimulants, I can mention one worth attention as not of unfrequent occurrence, and of great consequence to be aware of. A young female had been treated for two or three weeks by bleeding, neutral salts, and low diet, for what was called determination to the head, supervening upon what had been considered a pleuritic affection ; there was jactitation when I saw her, sense of oppression at the chest, incoherence of speech, severe pain of head occasionally causing her to put her hand to it, and to cry out, intolerance of light and sound, flushed face, but mark not fever, weakness but not sluggishness of the voluntary motions ; the pulse jerking as you find after hæmorrhage, but not firm ; the tongue not foul, but white as you frequently see with an empty stomach. I ascertained the pain at the first to have been in the *left* side, and from other circumstances, I felt satisfied that the present state was clavus hystericus kept up by inanition, she had been allowed the day before a little weak chicken broth, but her being supposed not able to bear it caused my being consulted ; wine and animal food gradually administered, without any medicine but a few drops of vinum ferri, soon calmed all the symptoms of what was called determination to the head, and health was restored in a few weeks.

These three cases will illustrate several points ; here we see, 1st, Local inflammation, and irritation, combined with fever which it has produced, lesion of the nervous system ; food and wine, affording no nourishment, narcotics no rest, cured by a sedative, digitalis.

2d, Local inflammation and irritation producing not fever (lesion of the nervous system) but constitutional irritation of it, chiefly evinced by loss of sleep, no indications for stimulants, sedatives, or tonics, and no want of strength or appetite, a narcotic by cutting off the communication with the nervous system gave it time to regain its natural state, and subsequently to give energy to the vessels of the inflamed part.

3d, Local inflammation and irritation (uterine), having produced, not fever, (lesion of the nervous system,) but irritation of it, constitutional irritation, hysteria : debility arising from want of nourishment, so that narcotics administered procured but temporary relief as giving no nourishment, and sedatives produce aggravation of the symptoms, stimulants and food by giving strength acted as a tonic, restoring power to the nervous system, and consequently to other parts.

Though keeping the bowels open is useful in chorea, and in a variety of nervous affections, to promote the digestion, &c. ; constitutional irritations, hysteria, &c., are always aggravated by much depletion, so that until food, and tonics give strength, there cannot be cure; now sometimes in constitutional irritation the patient cannot eat, any more than in fever, though from a different cause, sometimes there is no inclination for food from morbid delicacy of the senses of taste, and smell ; sometimes from irritability of the primæ viæ a mouthful produces a sense of repletion; and this nervous anorexia sometimes increases the difficulty of distinguishing between fever and irritation, and particularly when the primary local irritation is in the primæ viæ from indigestion, simulating gastroenteritis, inflammation of the mucous membrane." 117.

With the following passage containing our author's views of dropsy, we shall conclude this short notice of the work.

" We have now to enquire into the nature of Dropsy, that morbid state of the capillary exhalants which sometimes results from Inflammation ; and as Fever and Irritation though distinct affections, are frequently co-existing and complicated, so Dropsy is frequently combined with one or both of them.

Dropsy is an undue deposition of serous or watery fluid, by those capillary vessels of the serous membranes, and of the cellular tissue, which in a healthy state supply merely sufficient to keep those parts in a soft state.

Without for the present saying whether Dropsy be Inflammation or not, the proving or disproving of which proposition would not advance us in our investigation, I must observe, that the proximate cause of both is the same, a weakness and consequently giving way or enlargement of the capillary or minute arteries concerned ; this cause I hold to be sufficient for the explanation of the phenomena, and as I denied arterial action to be increased in inflammation, so I deny that it is necessary, as many do, to refer to a diminished action in the absorbents as a cause of dropsy. I consider the action of the absorbents to be uniform, and that, though there may be, it is not necessary to suppose any alteration as to absorption, as a cause either of inducing, or removing dropsical effusion ; for, considering the action of the absorbents to be uniform, merely to take up and carry off what is offered to them, it is evident that too great a quantity of fluid being poured out, the absorbents will not be equal to taking it up fast enough, but when we act upon the arterial capillaries, so as to check their exhalations, we know that, the absorbents, continuing their action, will gradually carry off the overplus of effused fluid ; and we know, that we can restrain the effusion from arterial capillaries, in two ways, either by constringing them, or by allowing less fluid to go to them ; but of the absorbents we know no demonstrable mode of altering their action, and I hold that the *medicines* which are commonly *said* to *increase the action* of the *absorbents*, act on the *capillary arteries* so as to check their deposition, and that this is the true account of the removal of dropsical swellings, by the action of mercury and other medicines, which cause either an alteration in the action of the capillaries, so as to stop their depositions, or by causing evacuation, actually diminish the quantity of matter supplied by the arteries, as Elaterium and diuretics do in dropsy, and Mezereon, Daphne Laureola and other drastic purgatives and emetics do in syphilis, when patients are cured without mercury by starving and sudorific and evacuant medicines.

Dropsy is not a disease in itself, a primary disease, it is only a constitutional disturbance, a symptom, a state induced by some diseased organ; the partial sudden effusions which sometimes take place from inflammation of a serous membrane such as the Empyema of Lænnec, from the pleura, (which by the bye is not the Empyema of Cullen) should rather be called Inflammatory Effusion than dropsy.

Dropsy is induced by any protracted disease which by irritation or slow fever robs the secretory organs of their nervous energy, as the kidneys, skin, and intestines, but above all the kidneys and skin, and when they cease to secrete the redundant fluid oozes from the overloaded capillaries, not merely overloaded but weakened, in consequence of the deteriorated state of the nervous system, and unless we can restore energy to the nervous system, so as to give tone to the exhalent capillaries, we in vain evacuate the fluid either by tapping, or by diuretics, or cathartics such as Elaterium.

Thus Dropsy is not to be considered as an isolated or single disease, except when, for a time, to prevent a patient from being overwhelmed by the fluid in the cavities, we turn our whole attention to getting rid of it, either by tapping, or profuse evacution, as by Hydragaque purgatives, or diuretics, and other medicines.

When we see a patient with dropsical swellings, our great object must be to cure the disease which produced the symptom of dropsy, dropsy being but a symptom.

It is true some cure both by attending to the one symptom alone, as when dropsy is the consequence of inflammatory disease of the lungs, pericardium, or liver, in which squills and digitalis are employed specifically as diuretics, with purgatives, to evacuate the fluid by the kidneys and bowels, and some form of mercury at the same time empirically; although the whole attention is given to the symptom of dropsy, yet the treatment is also applicable to the local affection; and in such a case, often the primary and secondary disease are cured together; and referring to local inflammation as the root of some dropsies, we can understand how in many cases, the abstraction of blood becomes a most useful assistant in the cure; as well as the advantage of bloodletting, when the heart and capillaries do not balance in power, for the mere object of taking off the injecting force of the heart, as for instance, when the specific diuretics fail from the congested state of the kidneys, venesection often proves the most powerful diuretic, as sometimes the most efficient cathartic.

On the other hand, in Dropsy of broken down constitutions, as well as in Inflammations with failure of the vital powers, by referring to the influence of the nervous system, in giving strength to the capillary system, so as to enable the capillary arteries to contract and resist tbe distending force of the heart, we can understand how, in some instance, stimulants as wine, by increasing the nervous energy, and consequently capillary action, may restrain the effusion, when evacuation would sink the patient; so that dropsy like inflammation is cured by opposite treatment, according to the state of the constitution." 128.

From the above extracts our readers will probably be induced to form a very favourable opinion of the author and his work.

